# Employee Experiences with a Newly Adopted Paid Parental Leave Policy: Equity Considerations for Policy Implementation

**DOI:** 10.1089/heq.2019.0007

**Published:** 2019-04-10

**Authors:** Dawn M. Richardson, Anna Steeves-Reece, Allea Martin, David A. Hurtado, Lisset M. Dumet, Julia M. Goodman

**Affiliations:** ^1^ Health Promotion & Community Health Program, OHSU-PSU School of Public Health, Portland, Oregon.; ^2^ Center for Health Research, Kaiser Permanente Northwest, Portland, Oregon.; ^3^ Oregon Institute of Occupational Health Sciences, Oregon Health & Science University, Portland, Oregon.; ^4^ Health Management & Policy Program, OHSU-PSU School of Public Health, Portland, Oregon.

**Keywords:** health equity, paid parental leave, policy implementation, qualitative data, socioecological theory

## Abstract

**Purpose:** Paid parental leave (PPL) policies offer immense opportunity to enhance health equity by providing financial stability to workers and promoting the health of families in the United States. Working in partnership with a local county government, which recently adopted a paid leave policy, we engaged in a qualitative substudy to enhance our understanding of how workers perceived and experienced the policy across levels of the socioecological framework.

**Methods:** Working in partnership with Multnomah County, a large public-sector employer in Portland, OR that recently adopted a PPL policy, we collected qualitative data through focus groups with employees. Data were transcribed, coded, and analyzed thematically.

**Results:** We conducted seven focus groups with county employees (*N*=35) in the fall of 2017. Three major themes emerged from the focus group data: intersectional inequities, disparities by department, and uneven benefits.

**Conclusions:** Our findings highlight the inequities of experience with the PPL policy across employees at individual, organization, and environmental levels. These findings offer insight and guidance for entities considering the adoption and implementation of such policies to consider concrete steps to enhance equity of access and experience.

## Introduction

Paid parental leave (PPL) policies provide employees with paid time off from work when they add a child to their family through birth, adoption, or foster placement. By ensuring that families maintain income during these times of transition, PPL policies can bolster economic stability, minimize financial stress, and facilitate a smooth return to work, particularly for women.^1–3^ These policies also promote health and well-being; PPL has been shown to decrease infant mortality,^4–8^ promote breastfeeding,^9^ and improve maternal mental health.^10–13^

Yet, despite these demonstrated benefits across the life course, paid leave is not mandated in the United States, the only high-income nation with no such policy. Only 15% of U.S.-based private-sector workers have access to paid leave through their employers.^14^ In the absence of mandated PPL, employees must cobble together an insufficient package of benefits, pulling from sick leave, paid and unpaid time off, and disability insurance to facilitate leave taking.

While the Family and Medical Leave Act (FMLA) provided important protection for workers, it is far from sufficient: employees at the lower end of the socioeconomic ladder, a significant percentage of which are people of color, are unable to take unpaid leave due to ineligibility or financial constraints.^15–17^ This reliance on employer-provided benefits amplifies socioeconomic inequities because generous benefit packages that include PPL are disproportionately provided by employers of more educated, privileged workers^18,19^ and available to those with higher incomes.^20^ Given the persistent health and socioeconomic inequities in the United States,^21–25^ the equity-enhancing potential of PPL is clear.^18,26^

Socioecological theory,^27^ which highlights how expanding spheres of influence (e.g., interpersonal, organizational, and environmental) interact to shape individual experiences and outcomes, is well suited as a conceptual framework to advance inquiry in this area. At the environmental level, enactment of a PPL policy—whether in the public or private sphere—promotes access to paid leave, and such policies are gaining momentum: to date, six states (California, New Jersey, Rhode Island, New York, Washington, Massachusetts) and the District of Columbia have passed paid family and medical leave laws, and a growing number of cities and counties have passed laws providing paid family or parental leave to government employees.^28,29^

The presence of a paid leave policy is one step toward promoting health equity by attempting to provide recipients (irrespective of gender, race, and ethnicity) with equal time off from work to spend with new additions to their families. Yet evidence suggests that PPL policies may not be accessible by or beneficial to all workers.^15,30^

The socioecological framework also highlights the role of organization-level factors, pointing to the relevance of supervisors and coworkers in facilitating or impeding work/family arrangements. Research on related family-friendly policies (e.g., flexible schedules, telework) has revealed the “hidden” costs associated with accessing these benefits, including supervisors' disapproval and coworker displeasure.^31^ At the interpersonal level, supervisors have been identified as a critical influence on how employees experience their workplace. Programs that improve family-supportive supervisor behavior have been linked to improved health and well-being for employees.^32,33^

With regard to individual-level factors, there remains limited evidence in the published literature detailing employee experiences, particularly using qualitative or ethnographic approaches, which are optimally suited for developing in-depth knowledge of individuals' lived experiences. While some qualitative work has been undertaken in this area (with U.S.-based participants), it has generally been restricted—in focus—to specific populations (e.g., families of children with special health care needs,^34^ fathers,^35^ dual career couples^36,37^) or particular aspects of leave taking (e.g., experiences with a second versus first child.^38^) Moreover, these studies do not directly address the ways in which employees interact with the benefits available to them to illuminate factors that could potentially improve their experiences.

To achieve maximum benefit from paid leave policies as they gain momentum across the country, it is critical to understand how these policies are interpreted and experienced by diverse employees. This clarification can help guide employers' equity efforts by identifying specific aspects of policy roll out that bolster (or inhibit) success. To enhance our understanding of how workers perceive and experience a newly adopted PPL policy, we conducted a research study in partnership with Multnomah County, a public sector employer in Portland, Oregon, with ∼5,000 regular (i.e., nontemporary) employees.

Multnomah County's PPL policy was adopted in November 2015 and provides up to 6 weeks of continuous or intermittent fully paid leave that can be used within 12 months of the birth, adoption, or foster placement of a child. Findings from the overarching study are forthcoming. In this article, we provide the in-depth findings from the qualitative portion of this research, which center employee perceptions of and experiences with this newly adopted PPL policy.

## Methods

### Recruitment and data collection

All regular benefits-eligible Multnomah County employees were eligible to participate in the overarching study. Employees were initially invited by email to participate in an online survey; employees who expressed interest in participating in follow-up focus groups were subsequently contacted through email and invited to participate. Focus groups occurred at three locations across the county, lasted 90 min, and were moderated by a female researcher from OHSU-PSU School of Public Health.

Our focus group guide included open-ended questions around: policy knowledge; decision-making and planning around leave-taking; workplace experiences; leave-taking among coworkers; and perceived policy impacts. Focus groups were audiorecorded with digital recorders. Participants provided written informed consent and received refreshments (coffee, snacks) for their time (due to the employer's rules regarding incentives for participation during paid work hours). The protocol was approved by the OHSU-PSU School of Public Health Institutional Review Board.

### Data analysis

Digital audio files were transcribed and checked for accuracy. Authors independently reviewed the seven transcripts to identify broad coding “bins.” We then engaged in applied thematic analysis^39^ as our analytic approach: while our research questions, guiding conceptual framework, and knowledge of existing literature were influential, our identified themes were generated inductively from the data itself.

Initial coding categories were refined into a codebook, which was subsequently uploaded—along with the transcripts—into Dedoose.^40^ The qualitative analysis team (first three authors) applied these codes to all transcripts, meeting regularly to discuss discrepancies and develop consensus. Upon coding completion, we engaged in an iterative process to raise overarching themes.

## Results

Thirty-five employees agreed to participate in a focus group in the fall of 2017 (Tables 1 and 2). We conducted five focus groups with employees that had recently added a child (*n*=24) and two focus groups with those who had not (*n*=11). The following three overarching themes emerged (Table 3).

**Table 1.** Sociodemographic Characteristics of Focus Group Participants (n=35)

	*n*	%
Gender		
Female	26	74.2
Male	6	17.0
Transmasculine	1	3.0
Gender expansive	1	3.0
Other	1	3.0
Race/ethnicity		
White	28	80.0
Black/African American	2	5.7
Latino	3	8.6
American Indian/Alaskan Native	1	3.0
Mixed	1	3.0
Household income		
$30,000 to $39,999	1	3.0
$50,000 to $59,999	5	14.3
$60,000 to $69,999	4	11.4
$70,000 to $79,999	6	17.1
$80,000 to $89,999	4	11.4
$90,000 to $99,999	1	3.0
$100,000 to $149,999	7	20.0
$150,000 or more	7	20.0
Educational attainment		
Some college (1–3 years)	6	17.0
College (4 years+)	14	40.0
Graduate school	15	42.9

**Table 2.** Workplace Characteristics of Focus Group Participants (n=35)

	*n*	%
Supervisory status		
Manager	8	22.9
Nonmanager	27	77.1
Department		
District Attorney's Office	1	3.0
Child and Human Services	9	25.7
Department of Community Justice	6	17.1
Department of Community Management	3	8.6
Department of Community Services	2	5.7
Health Department	9	25.7
Library	3	8.6
Sherriff's Office	1	3.0
Nondepartmental	1	3.0

**Table 3.** Key Themes: Illustrative Quotes

Theme	Representative statement
Experiences of inequity	“I would only take the amount of leave that I had paid, because I am the primary breadwinner in my family … I can't not work. Even though I had a C-section and that has an eight-week heal time, with the way our disability works, you will only get paid for up to six weeks. If this six-week option wasn't available, I would have [had] to come back to work within the first month.” (Female leave-taking employee)
	“I was on a rotation at a higher level than I was at, which is sort of like a test drive for whether you can do the job. Then I was taken out of that rotation as quickly as they could do it … so it is so obvious to me… that that factored into it.” (Female leave-taking employee)
Disparities by department	“If you are in a tiny unit … then they're just SOL [s^***^ out of luck]. People will resent you so there is pressure to maybe take less leave or be apologetic about it, or do whatever because now you feel like you are indebted to other people because you have decided to have a child and do what you are legally entitled to do.” (Female leave-taking employee)
	“I feel like that supervision piece can make it extremely inequitable for people to experience their parental leave. … I went through three supervisors in that process. I had one plan with the first supervisor, which was very understanding, a very generous plan … And then that person left the week I went on leave. And suddenly I had this new person, and they didn't want to honor the plan that I had in place.” (Female leave-taking employee)
	“There is an entity of the County, but it moves really slowly. It is not like a ship. It is more like an amoeba. There are some units that are like WAY out there [(her emphasis)], but the big blob of it is back here. … Going back to PPL, it is an attempt by leadership of the County to kind of move the whole thing in the family-friendly direction.” (Female employee)
	“It was a lot of paperwork and follow-up legwork, and that is not easy to do when you are pretty tired and distracted and not in the office and not tracking. It was not like I wasn't super appreciative for having the six weeks paid leave, but it just seemed difficult to manage when your brain is exhausted and out of touch.” (Female leave-taking employee)
Uneven benefits	“I really lucked out because the policy went into place four days before my son was born … I would have burned every bit of sick and vacation time that I had accrued. I would have had, at the end of that time, nothing to lean on in case there was a need.” (Male leave-taking employee)
	“They seem less frenzied. They seem ready to come back to work, and I think we need not lose site of the positive effect that it is having on … their ability to be at work and be present at work.” (Female employee)
	“I am not going to have kids, and I also would like six weeks of paid leave for stuff. I do have sick parents, and so … it feels like a little bit of a morale killer in an interesting way. I feel great about working at the County. … But I feel, in this one instance, it would be nice to figure out how we could extend a similar benefit to everyone.” (Female employee)

PPL, paid parental leave.

### Experiences of inequity

Participant experiences with leave taking reflected stark inequities by gender and financial standing. Female participants who identified as having limited income or being the sole earner in their household described unpaid leave as a nonoption, a situation reflected by this mother, who said: “Just because I am on maternity leave doesn't mean my bills take a break.”

Employees with more financial security, on the other hand, talked about maximizing all of their job-protected time to extend leave, even if it was unpaid: “I took all the leave I had legally, paid or unpaid. … I don't care about not having money. I just needed the time for myself.” In addition to the financial inequities, female-identified participants experienced leave taking differently due to adverse professional impacts (with subsequent economic implications), exemplified by one mother saying that she was “penalized for getting pregnant.”

Birth mothers described how childbirth and breastfeeding necessitated longer leave, particularly in the case of pregnancy complications or recovery from cesarean section. As one leave-taking participant pointed out, “It was so hard for me. It is still so hard to be the person who gave birth, and was breastfeeding, and then having to return to work full time.” While participants felt that paid leave made breastfeeding easier, they faced challenges with supply maintenance and insufficient pumping breaks upon return to work. One woman ultimately filed a harassment complaint due to treatment she experienced: “People were monitoring my time and telling management that I had more breaks than them. They said I wasn't working enough.” In other departments, however, female employees talked about colleagues going out of their way to make sure they felt comfortable pumping.

Participants further shared that race and ethnicity seemed to play a role in the dynamics of organizing and structuring leave. One female participant of color shared her experiences of inequity and how she has seen this replicated with other women of color: “… depending on the supervisor, someone gets something very generous and then a person in the next unit over gets not a lot. This is kind of horrible for all of us to see that, to see this inequity, even though we have these great policies.”

### Disparities by department

A salient theme across all focus groups was the view that workplace culture and environment play a major role in how the PPL policy is implemented and experienced by workers. Specifically, participants pointed to factors, including departmental size and resources, demographic makeup, and supervisor's attitude toward leave taking as influential for employee experience.

Understaffing of certain departments made employees feel guilty for taking time off, particularly when their leave might overburden coworkers. Beyond the scope of responsibilities, participants said departments and units all have their own leave-taking cultures, ranging from extremely accepting to judgmental and unsupportive. Participants used expressions like “old-fashioned” to describe the work environment of some departments. A female employee said, “I see the older generation resisting this more than the younger generation, especially in our department, just because it is male dominated.” In contrast, departments with more women and parents were described as more supportive. A woman who used the policy in departments unit said, “Everybody—my supervisors, they have kids, and grandkids—everybody was supportive.”

Participants further explained that supervisors have significant influence over the leave-taking experience. Unsupportive supervisors strongly and negatively influenced participants' experiences, planning and taking leave, and returning to work. With supportive supervisors, on the other hand, employees spoke of enhanced workplace trust, earlier planning of leave taking, more productivity before and after leave taking, and better collegiality. In addition, while some participants expressed gratitude for supervisors or department-level HR staff who helped them navigate the process of leave taking, many others said it was difficult to obtain concrete guidance.

### Uneven benefits

Both leave and nonleave takers highlighted various overlapping benefits of the PPL policy that centered around stress reduction, positive impacts on health and well-being, and facilitating an easier return to work. Participants repeatedly shared that had it not been for paid leave, their leave-taking experiences would have been characterized by stress and uncertainty. The policy was also credited with making return to work less stressful, with the capacity for making it easier to re-engage with work responsibilities and colleagues.

Agreeing with observations that those who had used the policy were more “mentally present,” upon return to work, one woman shared her view with this phrase: “Just being able to do your work and focus on that as opposed to being worried about your kid.” These and other similar remarks underscored the importance of paid leave for facilitating parents' ability to re-enter the workplace.

But while acknowledging the positive impacts of PPL and consistently expressing gratitude for the county's adoption of the policy, participants felt that there is much work to be done to arrive at a place where they truly see their employer as family friendly. Extending the leave to 12 weeks was a frequent suggestion, as were flexible work schedules or the option of working from home, the provision of free or low-cost childcare, and a more inclusive definition of family that accounts for aging parents or nontraditional adoptions.

Regarding this latter suggestion, a handful of participants highlighted their need to also care for loved ones who are not their children (e.g., close friends, siblings, aging parents). One employee suggested that employers' ideals on what constitutes “family” limits her ability to access these benefits: “If you are a person of color, if you are LGBTQ, if you are just a single woman—as we define family—[PPL] really doesn't pertain to you very much.”

## Discussion

This research makes two specific contributions to the extant body of literature on paid family leave policies in the United States. First, our results provide nuanced knowledge on how employees working for a large public employer perceive and experience the implementation of a newly adopted PPL policy. Second, this work highlights the inequity of experience across these employees at individual, organizational, and environmental levels. These findings offer insight and guidance for entities considering the adoption and implementation of such policies to consider concrete steps to enhance equity of access and experience.

Despite having equal access to PPL, participants felt that departmental characteristics and supervisor attitudes ultimately set the tone for the level of support offered to and experienced by leave-taking employees. They further described confusion and frustration resulting from their inability to gain clarity from supervisors or human resources personnel regarding the process of leave taking. The ability to secure correct and adequate guidance—and to satisfactorily navigate the leave-taking process—was described by our participants as being strongly influenced by the employee's gender, financial resources, job-related tasks, and supervisor's attitude toward leave taking and level of support for PPL.

In this study, our findings align with existing literature, where the importance of supervisor support to increase access and use of work/family arrangements is well-established.^32,33,41^ In addition, while our focus group findings demonstrated that participants take all available paid leave, regardless of perceived supervisor support, it was made clear that the length of leave does not fully determine the employee's experience. Participants raised equity concerns stemming from the leave-taking process: deciding to take leave; determining when to request leave; establishing a leave-taking plan with one's coworkers and supervisor; and returning to work. Participants also shared experiences of inequity related to the time away from work: difficult experiences planning for leave were related to increased stress while on leave and when returning to work.

In this area our findings also dovetail with an emerging discussion across multiple disciplines, highlighting the equity implications of upstream policies like PPL in the United States.^18,26,42–44^ Building on this literature, our results point to the need for supervisor training as a strategy for improving parental health and well-being. Additionally, given the importance of gender in our participants' PPL experiences, and the inability of our focus groups to tease these differences apart, we recommend that future studies do more to explore potential gender disparities.

Despite these challenges, participants generally agreed that adoption of the PPL policy signaled a commitment by their employer to move in a family-friendly direction. They spoke at length about the benefits observed and experienced as a result of the policy, including direct and indirect health benefits for mothers, children, and their families. This finding is in line with other qualitative work in this area, in which participants point to the positive impacts of paid leave on health and well-being.^34^

In the case of Multnomah County, it was due to the perception of the overwhelming benefit of PPL that our participants highlighted the need for additional family-friendly policies to extend the positive impacts stemming from the adoption of this specific policy. More workplace lactation support, affordable childcare options or subsidies, flexible schedules, and the ability to work from home were all mentioned as potentially beneficial policies.

Going further, participants expressed a strong desire for the definition of family to be expanded so that these policies could be accessed by a wider range of employees. In particular, many questioned the wisdom of limiting the new paid leave policy to parents while excluding other family caregivers. This echoes conversations in the policy and advocacy communities: the FAMILY Act being debated in Congress and all state paid leave policies include a range of family caregivers beyond new parents and, in some cases, have been amended over time to expand the definition of “family” for eligibility purposes.

Connecting our findings to our guiding conceptual framework, it is clear that influential factors at the individual (e.g., employee demographic characteristics), organizational (e.g., supervisor and/or departmental characteristics), and environmental (e.g., socially constructed spectrum of privilege and opportunity) levels independently and synergistically effect employee experiences with PPL. The adoption of the policy—seen by participants as a significant move toward a more family-friendly workplace—indicated progress and resulted in positive reception; however, the inequitable application of the policy (across individuals and departments) demonstrates the importance of adopting an equity lens in implementation and policy evaluation. Without a specific focus on equitable, upstream change that targets social and political aspects of the environments, in which all other levels of influence are shaped, our ultimate impacts of paid leave policies will be minimal.

### Limitations

Focus groups benefited from a diversity of perspectives when it came to age of participants, amount of time working at the County, leave- and nonleave takers, and birth and nonbirth parents. However, there were far more female than male participants and limited racial/ethnic diversity. Given findings on leave-taking disparities by demographics and socioeconomic status, there is a need to hear more about the nuances of leave-taking experiences across diverse employees.

### Health equity implications

Findings from this study offer guidance for employers considering adopting a PPL policy, as well as for policymakers and advocates who are developing paid leave policies in the public sphere. Results indicate two broad opportunities for policy improvement to reduce inequities: (1) policy implementation strategies developed and undertaken with an equity lens; (2) expanded policies to encourage family-friendly environments.

Training for supervisors and organizational leadership is one concrete strategy that could be used to promote equitable policy implementation. In addition to targeted training, supervisor training to enhance family-supportive behaviors could have a magnified impact by addressing the needs and concerns of diverse workers. Combined with policies like childcare subsidies, flexible scheduling, and the opportunity to telecommute, these strategies have the potential to encourage family-friendly environments and enhance work-life balance, and ultimately improve population health.

## Abbreviation Used

PPL: paid parental leave
